# Gestational diabetes knowledge improves with interactive online training modules: a pre-post analysis

**DOI:** 10.21203/rs.3.rs-2860961/v1

**Published:** 2023-06-02

**Authors:** Petra Krutilova, Roxann Williams, Rebecca Morey, Carole Field, Veronda Byrth, Melissa Tepe, Amy McQueen, Cynthia Herrick

**Affiliations:** Washington University School of Medicine, Metabolism & Lipid Research; Washington University School of Medicine, Metabolism & Lipid Research; TriStar Centennial Medical Center; Washington University School of Medicine, Metabolism & Lipid Research; Washington University; Affinia Healthcare; Washington University; Washington University School of Medicine, Metabolism & Lipid Research

**Keywords:** Gestational diabetes mellitus, diabetes education, online modules, nurses, community health workers

## Abstract

**Background:**

The risk of developing type 2 diabetes mellitus (T2DM) is up to 50% among women with gestational diabetes mellitus (GDM). GDM also increases risks for pre-term birth, macrosomia, fetal hypoglycemia, and C-section delivery. Education for expectant mothers with GDM about nutrition, exercise, and the risks of developing T2DM after delivery enhances the probability of postpartum diabetes screening. However, the availability of diabetes education is limited. To bridge this gap, our team developed four training modules on GDM tailored for nurses and community health workers. This pilot study assesses changes in knowledge, self-efficacy for providing diabetes education, attitudes, and intentions to recommend diabetes prevention before and after training completion.

**Methods:**

These interactive online modules, each lasting 45–60 minutes and featuring engaging case studies and integrated knowledge assessment questions, were disseminated through various professional organizations to clinical staff providing care for women with GDM. Optional pre- and post-training surveys were conducted to gauge the effectiveness of the modules. Collected data did not follow a normal distribution pattern. We provided an overview of the baseline characteristics of the population, self-efficacy, attitudes, intentions, and GDM knowledge by calculating the median scores and interquartile ranges. We assessed the changes in scores on self-efficacy, attitudes, intentions, and GDM knowledge before and after training using non-parametric Wilcoxon matched-pair signed rank tests.

**Results:**

Eighty-two individuals completed baseline evaluation and 20 individuals accessed all modules and completed post-training assessments. Among those completing the training, improvement was noted in GDM knowledge [56.5% (16.0) v. 78.3% (22.0), p < 0.001], Self-efficacy for providing diabetes education [6.60 (2.73) v. 9.33 (0.87), p < 0.001], attitudes toward the value of tight control [4.07 (0.79) v. 4.43 (0.86), p = 0.003], and intentions to recommend diabetes prevention measures [4.81 (0.63) v. 5.00 (0.00), p = 0.009)].

**Conclusions:**

Completion of our interactive online modules improved knowledge, intention to recommend diabetes prevention methods, self-efficacy to provide diabetes education, and attitudes toward the value of tight control among individuals caring for women with GDM. Enhanced accessibility to such curricula is crucial to improve access to diabetes education.

**Trial registration:**

This study was registered at clinicaltrials.gov, identifier: NCT04474795.

## Background

Gestational diabetes mellitus (GDM) is an important public health problem. A diagnosis of GDM increases the lifetime risk of maternal type 2 diabetes (T2DM) by 7-fold, and Black women have the highest risk for progression (1, 2). Furthermore, GDM increases the risk for maternal, obstetric, and fetal complications including macrosomia, gestational hypertension and pre-eclampsia, pre-term birth, fetal hypoglycemia, shoulder dystocia, and need for cesarean section (3). Impaired glucose metabolism also increases the risk of maternal obstetric and child developmental complications in future pregnancies (4). In addition, epidemiologic studies have demonstrated an association between the risk of obesity and T2DM in offspring of pregnancies complicated by GDM (5). Moreover, with national trends in increasing adolescent/young adult obesity, and age at first pregnancy, GDM rates will likely continue to rise.

Both diabetes self-management education and medical nutrition therapy are beneficial for glycemic control in GDM (6, 7). In fact, most cases of GDM (70–85%) can be managed with lifestyle modification alone (8). Postpartum diabetes screening at one year was higher among commercially insured women with GDM who saw a nutritionist or diabetes educator during pregnancy (9). In a nationwide postpartum sample, using Pregnancy Risk Assessment Monitoring System data, prenatal education on nutrition, exercise, and T2DM risk among women with GDM increased the likelihood of postpartum diabetes screening by 3-fold, and in a sample of women on Medicaid during pregnancy in Missouri, women who had a prenatal visit with a diabetes educator were 1.7 times more likely to receive postpartum diabetes screening (10, 11).

However, access to certified diabetes care and education specialists (CDCES) and registered dietitians is limited by lack of insurance coverage, inadequate physician referrals, provider scarcity, and challenges created by extra visits and copayments (12, 13). In under-resourced settings, nurses and other clinical staff may be expected to provide some of this education; however, these individuals may have variable levels of formal training and experience in this area. Inadequate knowledge of GDM management standards, attitudes about diabetes, and low self-efficacy for providing diabetes education may limit an individual’s ability to successfully provide this education.

Additionally, there are few freely available and accessible training modules on this topic for nurses and community health workers (CHWs). Our team found online educational resources for nurses; however, the majority of them were not available without charge (14, 15) or did not have the depth or breadth of information desired by our team (16). We were not able to find any training modules for CHWs. Additionally, we did not identify any literature evaluating the effect of these existing modules on learners’ knowledge or other measures.

To begin to address some of the education gaps in this area, our team created four online training modules for nurses and CHWs providing Maternal-Child Health care to under-resourced populations. We also adapted and designed surveys to evaluate the effectiveness of these training modules using measures of GDM knowledge, self-efficacy for providing diabetes education, diabetes attitudes, and intention to recommend diabetes prevention measures. The purpose of this pilot study was to evaluate whether completion of the training would significantly improve GDM knowledge, self-efficacy for providing diabetes education, diabetes attitudes, and intentions to recommend diabetes prevention measures.

## Methods

### Online training module development

The four training modules evaluated in this pilot study were created between July 2019 and June 2020. The intended audience for these modules was nursing and CHW staff providing Maternal-Child Healthcare at a community health center. The research team identified the need for modules that could be utilized at the learner’s pace with multiple opportunities to practice skills. Additionally, our team focused on creating example patient cases that would reflect racial and ethnic diversity of the patient populations served by community health centers. In the midst of module development, the COVID-19 pandemic began and the study team shifted to develop and deliver the modules online to a wider population of learners.

**Figure 1:**
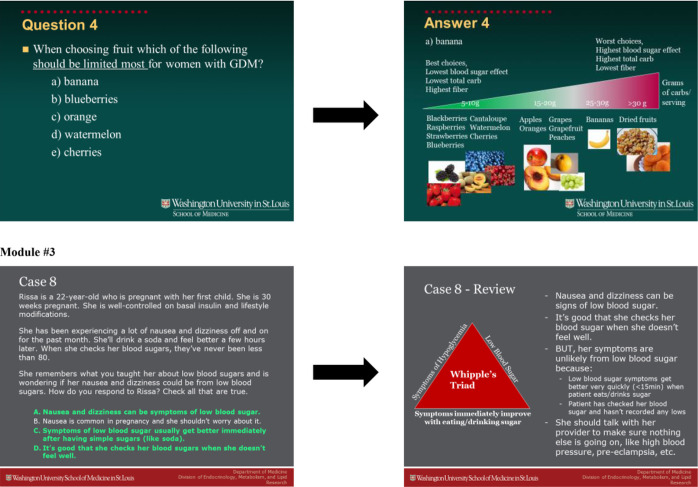
Examples of interactive components of teaching modules

The learning objectives for each online module are outlined in Table 1. These modules, lasting 45–60 minutes, consist of video presentations enriched with interactive and illustrative patient cases. Integrated within the modules is knowledge assessment that provided feedback to participants. Within each module, there are 4 to 9 patient cases as examples, specifically addressing key points of interest. Questions pertaining to these cases were integrated into the module’s flow, requiring participants to answer them in order to advance. Towards the end of each module, participants were presented with 5 to 6 knowledge assessment questions. Following each question, the correct answer along with an explanation was provided (Fig. 1, submitted separately).

Module #1 provides an introduction to GDM. It describes the changes in blood glucose metabolism in different stages of a normal pregnancy and compares them to abnormalities seen in GDM. It explains the risk factors for GDM, how to distinguish GDM from other types of diabetes, and describes how the diagnosis of GDM is made. This module also provides a summary of the consequences of uncontrolled diabetes for mother and baby. The focus of module #2 is non-medical management of GDM including nutrition and physical activity. It explains recommendations for weight gain and calorie intake during pregnancy as well as an introduction to macronutrient types. Strategies for creating a meal plan including cost concerns and grocery shopping tips are also part of this module. The last part of module #2 describes exercise recommendations during pregnancy and ideas for creating patient-centered physical activity plans. Module #3 describes blood glucose monitoring recommendations, blood glucose targets in GDM, and tips on how to interpret blood glucose logs. It explains different treatment options including insulin and other glucose-lowering agents. Description of different insulin types and their characteristics is included as well. This module also identifies the risks and common symptoms of hypoglycemia, and provides recommendations for hypoglycemia treatment. In module #4, the focus is on the future risk for T2DM in individuals with GDM. This module describes post-partum diabetes screening recommendations and challenges. It also provides ideas for patient-centered planning to minimize risks of diabetes development in the future including physical activity, healthy diet, and behavior modifications, focusing on the components of the Diabetes Prevention Program intensive lifestyle intervention and discussing the role of metformin and breastfeeding in diabetes prevention.

### Module dissemination and recruitment of participants

The training modules were hosted online and information regarding their accessibility was disseminated to clinical staff taking care of women with GDM through email, utilizing various professional organizations as the means of distribution. An email containing a concise introduction and a link to the modules’ website was sent to the contacts within the following organizations (Supplement 1): Nurse Family Partnership, MU Extension, St. Louis Regional Health Commission, St. Louis Integrated Health Network, Association of Women’s Health, Obstetrics and Neonatal Nurses, National Association of Community Health Workers, National Association of Community Health Centers, Missouri Nurses Association, Generate Health, the Missouri Diabetes Shared Learning Network, Missouri Primary Care Association, American Public Health Association, and St. Louis community college community health worker and nurse training programs.

Nursing continuing education credits (1 credit per module) were available via Washington University for module completion. On the modules’ home page, we offered information regarding the study, accompanied by optional pre- and post-training evaluations. Prior to completing of the pre-assessment forms, individuals were offered the option to participate in the study. Once the pre-assessment was finished, participants received a unique code to start the first module. Subsequently, at the conclusion of each module, a distinct code was assigned to proceed to the following module, finishing with the post-assessment after the last module. The employment of these codes ensured that participants accessed all modules prior to completing the post-assessment forms.

### Survey Measures

Pre-intervention data collection included measures to assess sample characteristics (age, race, ethnicity, degree/training, practice specialty). We also included measures to assess GDM knowledge, self-efficacy for providing diabetes education, diabetes attitudes, and intention to recommend diabetes prevention measures at both pre- and post-training. The GDM knowledge, self-efficacy for providing diabetes education, and intention to recommend diabetes prevention measures were developed for this study. Three subscales of the previously developed Diabetes Attitudes Scale (17) were also utilized. All scales are available in Supplement 2.

GDM knowledge was assessed with multiple choice questions designated for content covered in each training module. There were 23 questions divided into four indices, one for each module (Module 1: Questions 1–5, Module 2: Questions 6–11, Module 3: Questions 12–18, Module 4: Questions 19–23). We evaluated the percentage of correct answers for each module and all modules combined, both pre- and post-training.

The Self-Efficacy to Provide Diabetes Education scale includes 15 statements with response options that range from 1 (not at all confident) to 10 (totally confident) (Supplement 2). This scale was created for this study and modeled after the Lorig scale for self-efficacy with diabetes self-management (18). The response scale is identical to the Lorig scale, and the questions cover similar diabetes management domains as the Lorig scale. The scale demonstrated excellent internal consistency reliability in our pre-training sample (n = 68 with full data; Cronbach’s alpha = 0.94). Face and content validity were assessed through review of the scale by the study team and additional input from CDCES. Our sample was not large enough for factor analysis and full validation; however, preliminary assessment of construct validity demonstrated weak but positive correlations between this scale and the General Self-Efficacy Scale (GSES) (19) (n = 74; r = 0.11; p = 0.37) and the Physician Teaching Motivation Questionnaire Intrinsic Teaching Motivation Subscale (PTMQ) (n = 76; r = 0.29; p = 0.01) (20). Additionally, there was a positive correlation between the Self-Efficacy to Provide Diabetes Education score and GDM knowledge (n = 82; r = 0.26; p = 0.02). A mean of provided answers was utilized for the summary score on the Self-Efficacy to Provide Diabetes Education scale and median score was reported pre- and post-training.

Three subscales from the Diabetes Attitude Scale were assessed [patient autonomy (8 questions) (n = 77; a = 0.63), value of tight control (7 questions) (n = 77; a = 0.64), and need for special training (5 questions) (n = 78; a = 0.58)] (17). Responses were captured using a Likert scale from 1 (strongly disagree) to 5 (strongly agree). The summary score for each subscale is the mean of answered items. Median scores are reported pre and post training.

An additional scale created for this study, Intention to Recommend Diabetes Prevention Measures, included 8 questions (Cronbach’s alpha = 0.89). Items evaluated how likely respondents were to recommend specific behaviors to individuals with a history of GDM (i.e., postpartum diabetes screening in different time frames, breastfeeding, dietary modification, and physical activity). Responses were recorded on a Likert scale ranging from 1 (extremely unlikely) to 5 (extremely likely). Mean scores were utilized to represent intention. The stem and response options are similar to existing measures of intention, derived from the theory of planned behavior, which recognizes the variable or imperfect association between intention and behavior (21). The median summary score was reported pre and post training.

### Data Analysis

We examined descriptive statistics for all measures and time points. Data were not normally distributed and non-parametric tests were used for reporting and comparing pre-post training differences. We compared baseline characteristics of participants who accessed all 4 training modules (n = 20) to those who did not access all 4 training modules and assessments using the Mann-Whitney U test for continuous variables and chi-square test for categorical variables. For individuals missing some data in the calculation of scale scores, the mean of existing answers was used if more than 50% of the items in the scale were completed. Individuals missing demographic data or data for more than half of a subscale were excluded from those comparisons. The number of individuals with data missing for each comparison is indicated in Table 2 footnotes.

To assess the effectiveness of the modules, we evaluated changes in scores on GDM knowledge, Self-Efficacy to Provide Diabetes Education, Diabetes Attitudes Scale, and Intention to Recommend Diabetes Prevention Measures scales pre- and post-training among the 20 individuals who completed the training, using Wilcoxon matched pair signed-rank tests.

Finally, we evaluated adoption of the modules and retention in the overall training by evaluating the percentage of individuals accessing each module and the time frame during which most participants accessed all four modules and completed pre- and post-training assessments. These data were obtained from electronic data captured by the module software with a unique date and time stamp for each login. Study participants were prompted to input their unique ID on pre- and post-assessments and when starting each module so that retention could be assessed.

All analyses were conducted using SPSS v. 28 (IBM, Armonk, NY). As data collected were not identifiable, brief information was provided to participants about the program evaluation and no formal consent for participating was required. The study was approved by the Washington University Human Research Protection office on July 9, 2020 with a waiver of written informed consent; IRB number 202007060. Individuals who agreed to participate in the study and accessed all 4 modules and pre- and post-assessments within a 3-month time frame were given the opportunity to enter a drawing for $40 Visa gift cards.

## Results

A total of 85 individuals answered at least one question on the baseline survey. Three individuals were excluded because they did not answer > 50% of the items for any of the baseline measures. We analyzed 82 surveys for evaluation of baseline characteristics. The median age of respondents was 37.0 years (IQR = 19.0) and 98.8% identified as female. In terms of race and ethnicity, 64.6% identified as white non-Hispanic, followed by 17.1% Black or African American, and 9.8% Latinx or Hispanic. The majority (56.0%) of participants were nurses and dietitians, 22.0% were CHWs or medical assistants, and 22.0% reported other forms of education/training including social work, case management, nurse practitioner, lactation consultant, parent educator, and public health. The most commonly reported specialty was Obstetrics-Gynecology and Maternal-Child Health representing 63.4% of baseline participants. Internal Medicine and Family Medicine represented 8.5% of participants and the remaining 28.0% of participants came from disciplines such as public health, social work, community health, pediatrics, child development, and hospital settings.

Of the 82 individuals with sufficient pre-assessment data, 65 (79%) accessed module #1, 45 (55%) accessed module #2, 41 (50%) accessed module #3, and 20 (24%) accessed module #4 and completed the post-assessment. An additional 6 (7%) individuals completed the pre-assessment and accessed all 4 modules, but did not complete a post-assessment. Attrition was greatest between modules 3 and 4. Of the 20 individuals who completed the post-assessment, median time from start of the pre-assessment to end of the post-assessment was 11 days (IQR 20). Fifty five percent of the completer population finished both assessments and the training within 14 days and an additional 30% had finished within 30 days. There were 2 outliers (10%) among the completers who took more than 90 days to complete the training and pre- and post-assessments.

Twenty individuals completed both pre- and post-assessments and accessed all 4 training modules. Individuals with Obstetrics-Gynecology or Maternal-Child Health specialty were significantly more likely to complete the training (90% of completers versus 55.4% of non-completers had this specialty; p = 0.02). We did not find any other significant differences between completers versus non-completers on age, gender, race/ethnicity, education/training or baseline knowledge, self-efficacy, attitudes, and intentions (Table 2).

### Bold p value = 0.02 (Fisher exact test)

^1^ Missing = 1 (Completer); ^2^ Missing = 2 (Non-completers); ^3^ Missing = 5 (1 Completer; 4 Non-completers); ^4^ Missing = 4 (Non-completers); ^5^ Missing = 7 (Non-completers)

Among training completers, GDM knowledge overall improved significantly post- training. Median pre- to post-module knowledge scores increased from 56.5% (IQR 16.0) to 78.3% (IQR 22.0) (p < 0.001). The biggest improvement in knowledge was noted in the subscale assessing module #3 which focused on monitoring of GDM, medications, and hypoglycemia prevention (median score increased from 50.0% (IQR 39.0) to 71.4% (IQR 14.0), p < 0.001).

Self-Efficacy to Provide Diabetes Education also improved from pre- to post-training with median scores increasing significantly, from 6.60 (IQR 2.73) pre-training to 9.33 (IQR 0.87) post-training (p = < 0.001).

Analysis of Diabetes Attitude Scale scores revealed improvement in only one of the three subscales. Individuals had a more positive attitude toward the value of tight blood sugar control in GDM post-training compared with pre-training. Median scores increased from 4.07 (IQR 0.79) to 4.43 (IQR 0.86) (p = 0.003). Scores for the subscale assessing attitudes about patient autonomy and the need for special training remained unchanged pre- to post-training.

Scores on the Intention to Recommend Diabetes Prevention Measures scale increased after training with median scores increasing from 4.81 (IQR 0.63) pre-training to 5.00 (IQR 0.00) post-training (p = 0.009).

A score comparison on multiple scales before and after training among individuals who completed all four modules before and after training is summarized in Table 3.

## Conclusions/discussion

This pilot study demonstrates that individuals completing our interactive online modules show significant improvement in their GDM knowledge, self-efficacy to provide diabetes education, attitudes about the value of tight control, and intention to recommended diabetes prevention measures. We believe that such curricula are critical to improve access to diabetes education for nurses and CHWs providing care for women with GDM. In under-resourced settings, patients may not be able to meet with a CDCES or registered dietitian during pregnancy because of lack of insurance coverage, provider scarcity, and difficulty getting to visits or multiple copayments, so additional accessible education for existing clinical staff is essential as these individuals may be responsible for assisting patients with GDM management.

Nurse-led diabetes self-management education improves lifestyle, clinical, and psychosocial outcomes among patients (22). Therefore, novel methods to increase knowledge and self-efficacy for providing diabetes education among nurses and CHWs are critical. Virtual education is an easily accessible alternative to a traditional in-person approach and is well accepted by learners. It has been found to be effective for different levels of learners and with various delivery mechanisms. This pilot study assesses a novel online GDM training program focused on CHWs and nurses, but our findings are in agreement with similar studies on type 1 and type 2 diabetes-related education from other centers in the US and worldwide (23–30). Several studies have shown that distance training programs for CHWs and nurses – either combined with some in-person sessions (23, 28) or purely virtual (25, 29) – improve knowledge, confidence, and attitudes about providing care to patients with diabetes. Two recent studies demonstrated that virtual training is feasible and acceptable for delivering diabetes education to large groups of school-based nurses and other personnel (27, 30). Programs conducted with school personnel and CHWs in rural and remote areas rely on virtual education methods (24, 26) highlighting its advantages including reach, accessibility, and lower cost. Nonetheless, in-person education may still result in higher knowledge acquisition than virtual learning. A study comparing e-learning with in-person education on diabetic foot care among nurses showed improvement in knowledge in both groups; however, participants who completed in-person workshops achieved higher knowledge scores than those attending virtually (31).

Our study has several limitations. Unfortunately, the percentage of participants who accessed all modules and completed pre- and post-training assessments was low (24%). Several factors most likely contributed to the overall low completion rate. We started distributing modules in the midst of the COVID-19 pandemic, a very challenging period for healthcare workers, when GDM education was not one of the top priorities in health care. Another limiting factor might be that, in order to disseminate to the broadest possible audience, we included individuals from various specialties and backgrounds that self-identified a role in caring for women with GDM. Individuals with social work, public health, and case management backgrounds may not have found the modules to be as directly relevant to their work, lowering their likelihood of completing the series. This is supported by the striking difference in the retention by specialty. We noted that participants from Obstetrics-Gynecology and Maternal-Child Health were most likely to complete training and assessments, whereas completion rates for other specialties was lower (34.6% completed from Obstetrics-Gynecology and Maternal-Child Health versus 14.3% from Family Medicine/Internal Medicine versus 4.3% from other specialties). Finally, because we wanted to maintain anonymity of the surveys, we did not collect contact information and we did not use any reminders to prompt participants to initiate their training or remind them to complete modules once they started. We noted that the majority of individuals who completed the modules (85%) did so within a period of 30 days and 55% completed the modules within 14 days.

Despite these limitations, our study clearly demonstrated that interactive online training modules represent an easily accessible and effective way of improving GDM knowledge, self-efficacy for providing diabetes education, attitudes about the value of tight glucose control, and intention to recommend diabetes prevention measures to women with GDM. Information about retention and the timeline of completing modules from this pilot study will guide further dissemination plans for this training. For example, future dissemination will focus delivery to Obstetrics-Gynecology and Maternal-Child Health practitioners and incorporate incentives and reminders to complete training within 1 month.

Further dissemination and implementation of this online training program will be guided by implementation science methodology. A hybrid effectiveness-implementation study, guided by both implementation process (CFIR) and outcomes (RE-AIM) frameworks, could further evaluate the real-world effectiveness of the training and assess its implementation in different contexts (32, 33). Additionally, incorporating specific behavior change strategies could bolster the effect of this training on patient outcomes (34). Stakeholders could include Medicaid managed care plans who employ nurse case managers and CHWs to engage with members during and after pregnancy. These plans are highly motivated to improve health outcomes among their enrollees and may have training infrastructure built for their employees to obtain continuing education in which this training could be integrated. Additional stakeholders include nursing and CHW training programs, community health centers, hospital-based high risk pregnancy programs, home visiting programs, and others. Patient education materials that could be disseminated in print and online have also been developed which align with topics covered in the online training for nurses and CHWs. An evaluation of these materials will be published separately; however, future studies could assess online staff training and patient education as a multi-level intervention with different implementation strategies to improve self-management during pregnancy and adoption of diabetes screening and prevention measures in the post-partum period. In the post-COVID landscape, creative use of online training modalities remains essential to expand access to diabetes education among relevant healthcare stakeholders.

## Figures and Tables

**Table 1:** Learning objectives of online training modules

Module #	Topic	Learning objectives
1	GDM Disease, Diagnosis and Complications	Define gestational diabetes (GDM) & how it is diagnosed in the United States. Compare changes in blood sugar control during normal pregnancy and GDM. Identify risk factors for GDM. Summarize the consequences for uncontrolled GDM for mom & baby.
2	Nutrition and Physical Activity Management	Define recommendations for weight gain during pregnancy. Describe types of nutrients and examples of each. Apply strategies for meal planning including cost concerns. Design alternative plans for different dietary preferences. Explain exercise recommendations during pregnancy. Create a patient-centered trimester-specific physical activity plan.
3	Monitoring, Medications, and Avoiding Hypoglycemia	Describe blood sugar monitoring recommendations and goals in women with GDM. Identify the risks and common symptoms of hypoglycemia and determine appropriate treatment for these events. Explain GDM treatment options including recommended medications.
4	Future Diabetes Risk and Prevention	Assess patienťs risk of future diabetes mellitus. Describe current postpartum diabetes screening recommendations. Construct a patient-centered plan to minimize risks of future diabetes mellitus.

**Table 2:** Baseline sample characteristics

Characteristic	All N = 82	Completers N = 20	Non-completers N = 62
**Demographics**	**n (%)**		
Gender identity (Female)	81 (98.8)	20 (100)	61 (98.4)
Race/ethnicity			
Black or African American	14 (17.1)	5 (25.0)	9 (14.5)
Latinx or Hispanic	8 (9.8)	3 (15.0)	5 (8.1)
Native American, Asian, another race	7 (8.5)	0 (0.0)	7 (11.3)
White, non-Hispanic	53 (64.6)	12 (60.0)	41 (66.1)
Education/Training			
Nurses (RN/LPN/CHN), CDCES or RD	46 (56.0)	12 (60.0)	34 (54.8)
CHW or MA	18 (22.0)	4 (20.0)	14 (22.6)
Other	18 (22.0)	4 (20.0)	14 (22.6)
Specialty			
Internal Medicine/Family Medicine	7 (8.5)	1 (5.0)	6 (9.7)
Ob-Gyn/Maternal-Child Health	**52 (63.4)**	**18 (90.0)**	**34 (54.8)**
Other	23 (28.0)	1 (5.0)	22 (35.5)
	**Median (IQR)**		
Age (years)^1^	37.0 (19.0)	35.0 (20.0)	38.5 (17.0)
**Pre-test scores**			
GDM knowledge (max. score 100)^2^			
Overall	56.5 (17.0)	56.5 (16.0)	56.5 (17.0)
Module 1	60.0 (20.0)	60.0 (35.0)	60.0 (20.0)
Module 2	66.7 (17.0)	66.7 (17.0)	66.7 (29.0)
Module 3	57.1 (14.0)	50.0 (39.0)	57.1 (14.0)
Module 4	40.0 (20.0)	60.0 (35.5)	40.0 (40.0)
Self-Efficacy to Provide Diabetes Education Scale (max. score 10) ^3^	7.00 (2.37)	6.60 (2.73)	7.10 (2.27)
Diabetes Attitudes Scale (max. score 5) ^4^			
Patient autonomy	4.25 (0.53)	4.44 (0.84)	4.25 (0.50)
Value of tight control	4.14 (0.86)	4.07 (0.79)	4.14 (0.86)
Need for special training	4.80 (0.40)	5.00 (0.40)	4.80 (0.40)
Intention to Recommend Diabetes Prevention Measures Scale (max. score 5) ^5^	4.63 (0.88)	4.81 (0.63)	4.63 (0.88)

IQR = interquartile range; RN = registered nurse; LPN = licensed practical nurse; CHN = community health nurse; CDCES = certified diabetes care and education specialist; RD = registered dietitian; CHW = community health worker; MA = medical assistant

Bold p value = 0.02 (Fisher exact test)

1 Missing = 1 (Completer)

2 Missing = 2 (Non-completers)

3 Missing = 5 (1 Completer; 4 Non-completers)

4 Missing = 4 (Non-completers)

5 Missing = 7 (Non-completers)

**Table 3:** Score comparison on multiple scales before and after training among individuals who completed all four modules and pre/post assessments

Characteristic	Pre- training N = 20	Post- training N = 20	p value
Median (IQR)			
GDM knowledge (max. score 100)			
Overall	56.5 (16.0)	78.3 (22.0)	**<0.0001**
Module 1	60.0 (35.0)	80.0 (35.0)	0.172
Module 2	66.7 (17.0)	83.3 (17.0)	**0.013**
Module 3	50.0 (39.0)	71.4 (14.0)	**<0.0001**
Module 4	60.0 (35.5)	80.0 (20.0)	**0.002**
Self-Efficacy to Provide Diabetes Education Scale^1^ (max. score 10)	6.60 (2.73)	9.33 (0.87)	**<0.0001**
Diabetes Attitudes Scale (max. score 5)			
Patient autonomy	4.44 (0.84)	4.38 (0.72)	0.606
Value of tight control	4.07 (0.79)	4.43 (0.86)	**0.003**
Need for special training	5.00 (0.40)	5.00 (0.35)	0.475
Intention to Recommend Diabetes Prevention Measures Scale (max. score 5)	4.81 (0.63)	5.00 (0.00)	**0.009**

SD = standard deviation; IQR = interquartile range

Pre- and post-training compared using Wilcoxon paired signed rank test.

1 Missing = 1 (did not complete > 50% of items on pre-training scale)

## Data Availability

All data generated or analyzed during this study are available from the corresponding author on reasonable request.

## Abbreviations

CDCES: certified diabetes care and education specialists
CHW: community health worker
GDM: gestational diabetes mellitus
GSES: eneral self-efficacy scale
IQR: interquartile range
T2DM: type 2 diabetes mellitus
